# Association between meniscal volume and development of knee osteoarthritis

**DOI:** 10.1093/rheumatology/keaa522

**Published:** 2020-09-25

**Authors:** Dawei Xu, Jan van der Voet, Nils M Hansson, Stefan Klein, Edwin H G Oei, Femke Wagner, Sebastia M A Bierma-Zeinstra, Jos Runhaar

**Affiliations:** 1 Department of General Practice, Erasmus MC University Medical Center Rotterdam, The Netherlands; 2 Department of Radiology & Nuclear Medicine, Erasmus MC University Medical Center Rotterdam, The Netherlands; 3 Department of Medical Informatics, Erasmus MC University Medical Center Rotterdam, The Netherlands; 4 Department of Orthopedics, Erasmus MC University Medical Center Rotterdam, The Netherlands

**Keywords:** meniscal volume, knee osteoarthritis, MRI

## Abstract

**Objective:**

To assess the association between meniscal volume, its change over time and the development of knee OA after 30 months in overweight/obese women.

**Methods:**

Data from the PRevention of knee Osteoarthritis in Overweight Females study were used. This cohort included 407 women with a BMI ≥ 27 kg/m^2^, free of OA-related symptoms. The primary outcome measure was incident OA after 30 months, defined by one out of the following criteria: medial or lateral joint space narrowing (JSN)  ≥ 1.0 mm, incident radiographic OA [Kellgren and Lawrence (K&L)  ≥ 2], or incident clinical OA. The secondary outcomes were either of these items separately. Menisci at both baseline and follow-up were automatically segmented to obtain meniscal volume and delta-volumes. Generalized estimating equations were used to evaluate associations between the volume measures and the outcomes.

**Results:**

Medial and lateral baseline and delta-volumes were not significantly associated to the primary outcome. Lateral meniscal baseline volume was significantly associated to lateral JSN [odds ratio (OR) = 0.87; 95% CI: 0.75, 0.99], while other measures were not. Medial and lateral baseline volume were positively associated to K&L incidence (OR = 1.32 and 1.22; 95% CI: 1.15, 1.50 and 1.03, 1.45, respectively), while medial and lateral delta-volume were negatively associated to K&L incidence (OR = 0.998 and 0.997; 95% CI: 0.997, 1.000 and 0.996, 0.999, respectively). None of the meniscal measures were significantly associated to incident clinical OA.

**Conclusion:**

Larger baseline meniscal volume and the decrease of meniscal volume over time were associated to the development of structural OA after 30 months in overweight and obese women.

**Rheumatology key messages**

- Medial and lateral baseline volume were positively associated to K&L incidence, while medial and lateral delta- volume were negatively associated to K&L incidence.
- Lateral meniscal baseline volume was associated to lateral JSN.

## Introduction

The diagnosis of OA is mainly based on symptoms and radiographic features. Since 1986, ACR criteria have been used to classify knee OA [1]. More recently, MRI has been shown to have a higher sensitivity in detecting structural knee OA, especially when compared with Kellgren and Lawrence (K&L) grading on weight-bearing posterior-anterior flexed knee radiographs [2]. Several studies indicated that MRI is able to detect early OA features in asymptomatic persons without radiographic knee OA [3, 4]. Radiographic abnormalities in OA have been described extensively, including joint space narrowing (JSN), sclerosis of subchondral bone and the presence of osteophytes. Compared with the surrogate measurement of JSN on radiographic images, MRI enables direct evaluation of the cartilage, which is the main abnormality in OA. Therefore, the MRI holds promise as an alternative to radiography in the evaluation of joint structure [5], although until now there has been no consensus or a standardized scoring system for knee OA, especially in quantitative MRI-based measurement.

It is widely accepted that a strong causal relationship between meniscal damage and structural progression of OA exists [6]. A meniscal pathway to knee OA was implicated by a loss of meniscal function due to damage or extrusion, leading to increased biomechanical stress in the knee joint. This stress results in damage such as cartilage loss, subchondral bone changes, bone marrow lesions and synovitis, eventually resulting in symptomatic OA [7]. In view of this significant pathway in the pathogenesis of OA, it is important to assess the presence of meniscal pathologies, especially when studying early-stage knee OA.

To better understand the meniscal changes, previous studies described meniscal constructs such as volume, extrusion, thickness (height) and tibial coverage [8–10]. In a recent study, we confirmed an independent association between meniscal extrusion and the development of knee OA in overweight and obese women [11]. However, extrusion was scored semi-quantitatively using MRI Osteoarthritis Knee Score (MOAKS) [12], which does not consider the absolute sizes of both tibial plateau and meniscus, and the percentage of tibial cartilage covered by the meniscus.

The quantification of meniscal volume has been explored by segmentation of MRI images to obtain 3D volumetric morphometry. However, until now, there are still conflicting results on the association between meniscal volume and incident knee OA [13–15]. In this study, we therefore evaluated the association between both baseline meniscal volume and its longitudinal change and incident knee OA among middle-aged, overweight and obese women. By quantitatively analysing meniscal volume for those who are at high risk for OA development, we tried to determine whether meniscal volume could be a biomarker for incident knee OA.

## Methods

For this study, data from the PRevention of knee Osteoarthritis in Overweight Females (PROOF) study [16] were used. Details regarding this study have been described previously (ISRCTN 42823086) [14]. In short, the original study was a randomized controlled trial in which the intervention groups received a weight-loss programme and/or glucosamine sulphate or placebo, to determine whether these interventions prevent the onset of knee OA. As both interventions proved to have no significant effects on OA development, data is here treated as a cohort, with additional adjustments for the randomized intervention groups. The PROOF study has been approved by the Medical Ethics Committee of Erasmus MC University Medical Center Rotterdam, the Netherlands.

### Subjects

This cohort consisted of 407 overweight and obese women between 50 and 60 years old with a BMI ≥ 27 kg/m^2^. At baseline they were free of symptoms of knee OA according to the clinical criteria of the ACR [17] or other rheumatic diseases, were not being treated for knee complaints, were not using walking aids, had no contraindications for MRI, mastered the Dutch language, and did not use glucosamine [16, 18]. The participating women were recruited through their general practitioner. At both baseline and 30 months follow-up (FU) time, all subjects filled in a questionnaire on knee pain, physical activity level, quality of life, previous knee injuries, menopausal status and comorbidities. They also underwent a physical examination for Heberden’s nodes and measurement of body weight and height to calculate the BMI at baseline and FU.

### MRI and radiography

MRI (1.5 T) was performed using the Philips Medical Systems (Model Intera), SIEMENS (Model Symphony and Model MAGNETOM ESSENZA) with a dedicated rigid knee coil for all knees at baseline and after 30 months FU. The protocol included coronal and sagittal non-fat suppressed proton density-weighted sequences (slice thickness 3.0 mm/slice gap 0.3 mm) and a sagittal 3D water selective sequence (WATS) with fat saturation (slice thickness 1.5 mm) with a coronal planar reconstruction, amongst other sequences [18]. Meniscal pathology, including extrusion and tears, was scored on the MR images by two trained readers and an experienced musculoskeletal radiologist, using the MOAKS scoring system [12, 19]. As previously published, the reliability of the scoring of the change in MOAKS features, determined by prevalence-adjusted bias-adjusted kappa (PABAK) statistics, showed ‘substantial’ to ‘nearly perfect’ agreement (range 0.77–0.88, observed agreement 89–94%) [19, 20].

Weight-bearing semi-flexed posterior-anterior knee radiographs of both knees were acquired with the metatarsophalangeal protocol [21] at baseline and after 30 months and scored according to the K&L criteria [22]. Joint space width and the medial knee alignment angle were measured on the radiographs for all knees. As previously described, reproducibility tests showed moderate agreement for KL grade (κ  = 0.6) and good agreement for alignment (κ  =  0.7) and minimal joint space width (κ  =  0.7) [16].

### Meniscus segmentation and volume quantification

The medial and lateral menisci from all knees at baseline and FU were segmented fully automatically in the coronal, proton density-weighted MRI scan, using in-house developed software that combines multi-atlas segmentation-by-registration with a high-dimensional voxel-based appearance model [23–25]. In this approach, the atlas was formed by 25 MRI scans from the PROOF data, which were manually segmented by using open-source ITK-SNAP software [26]. Manual segmentation of the menisci was performed on the coronal proton density sequence and was checked on the sagittal proton density and sagittal WATS images. Segmentation was done from anterior to posterior and performed on all slices where the meniscus was identifiable.

After the baseline and FU meniscal volumes were acquired from the segmentation, volume change over time (delta-volume) and relative volume change (relative delta-volume) were calculated. Delta-volumes were calculated by subtracting the baseline volume from the FU volume. The relative delta-volume was obtained by expressing the delta-volume as a percentage of the baseline volume, positive changes of volume over time signifying growth of meniscus, while negative changes signify shrinkage.

### Outcome measures

The primary outcome measure was the incidence of knee OA after 30 months, which was defined for each knee as at least one out of the following three criteria: (i) JSN in the medial or lateral compartment  ≥ 1.0 mm; (ii) incident radiographic knee OA, defined by K&L  ≥  2 at FU, with baseline K&L < 2; or (iii) incident clinical knee OA according to the combined clinical and radiographic ACR criteria. The secondary outcomes were any of these items separately.

### Statistics

Descriptive statistics were used for the baseline characteristics. To verify the reliability of the automated meniscus segmentation on MRI, we performed a 10-fold cross-validation [27] experiment on the atlas set of 25 MRI scans, comparing the automatic segmentations with the manual segmentations using the Dice similarity coefficient (DSC) [28]. The value of DSC ranges from 0, indicating no spatial overlap between the two segmentations, to 1, indicating perfect agreement [28]. The association between independent variables [baseline and (relative) delta-volumes] and both primary and secondary outcomes were analysed separately. These analyses were done by performing generalized estimating equations (GEE) in SPSS 25, which treated two knees within subjects as repeat measurement. The GEEs were adjusted for baseline meniscal volume of medial or lateral side (when using baseline volume as independent factor, using 100 mm^3^ as a unit), medial or lateral delta-volume (when using delta-volume as independent factor, using 100 mm^3^ as a unit), BMI, age, knee injury, knee alignment, postmenopausal status, Heberden’s nodes, meniscal pathologies, meniscal extrusion, osteophytes and cartilage defects at baseline. Also, to further understand the relationship between meniscal volume and meniscal extrusion, we analysed whether meniscal volume was a confounder for the previously published association between meniscal extrusion and OA development in the same cohort [11]. A *P*-value <0.05 was used to indicate statistical significance in all tests.

## Results

### Baseline and FU characteristics

A total of 407 women were eligible to participate in the PROOF study. First, 97 knees without MRI data at baseline were removed. In addition, knees with missing data for the primary outcome (*n* = 91) were excluded leaving 626 knees (338 subjects) for the final analysis. There were no statistically significant differences in baseline characteristics between included and excluded knees (data not shown). All baseline characteristics of the eligible sample are presented in Table 1.

**Table 1:** Characteristics and features of the knee joint at baseline

Characteristic variables	*N* (%)	Mean (s.d.)
Age at baseline (yr)	814 (100)	55.7 (3.2)
Baseline BMI (kg/m^2^)	814 (100)	32.4 (4.3)
Baseline self-report knee injury	101 (12.4)	
Baseline cartilage defect	411 (50.5)	
Baseline osteophyte	474 (58.2)	
Heberden’s nodes	216 (26.5)	
Knee varus alignment	323 (39.7)	
Baseline postmenopausal	550 (67.6)	
Meniscus pathologies without extrusion	504 (61.9)	
Baseline medial volume (mm^3^)	723 (88.8)	1343.21 (320.50)
Baseline lateral volume (mm^3^)	721 (88.6)	1129.99 (263.17)
Baseline medial meniscal extrusion	203 (24.9)	
Baseline lateral meniscal extrusion	18 (2.2)	
K&L scores	810 (100)	
K&L = 0	412 (50.9)	
K&L = 1	344 (42.5)	
K&L = 2	49 (6.0)	
K&L = 3	5 (0.6)	
Clinical knee OA	32 (4.0)	

Baseline meniscal extrusion was defined as MOAKS ≥ 2, Heberden’s nodes was defined as a Heberden’s node in at least one hand. K&L: Kellgren and Lawrence.

One hundred and eleven knees (17.7%) developed knee OA according to the primary outcome after 30 months. Thirty-three knees (5.3%) developed medial JSN, 36 knees (5.8%) developed lateral JSN, 72 knees (11.6%) developed incident radiographic knee OA, and 49 knees (7.8%) developed incident clinical knee OA.

### Meniscus segmentation

**Fig. 1:**
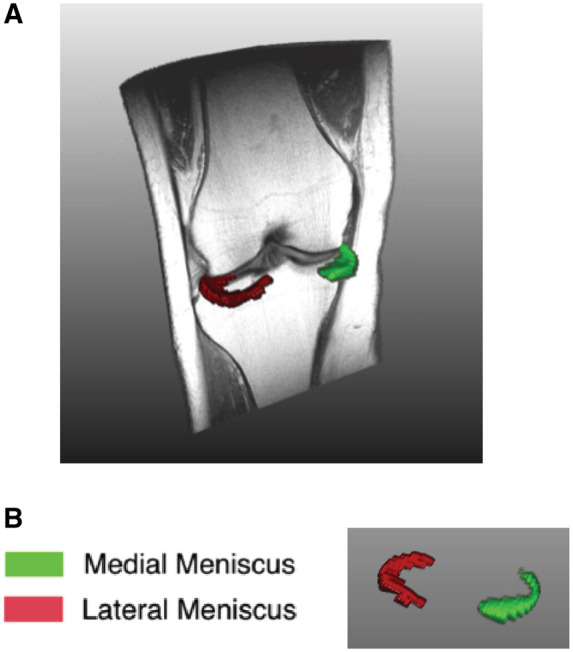
Example of meniscus segmentation (**A**) 3D overview of one left knee and coronal view of meniscus segmentation. (**B**) 3D view of meniscus from segmentation (green: medial meniscus; red: lateral meniscus).

An example of meniscus segmentation was shown in Fig. 1. The cross-validation experiment on the atlas resulted in an average DSC of 0.75, which is in line with results reported in the literature for automated meniscus segmentation on 1.5 T MRI [29, 30].

### Baseline meniscal volume and knee OA development

**Fig. 2:**
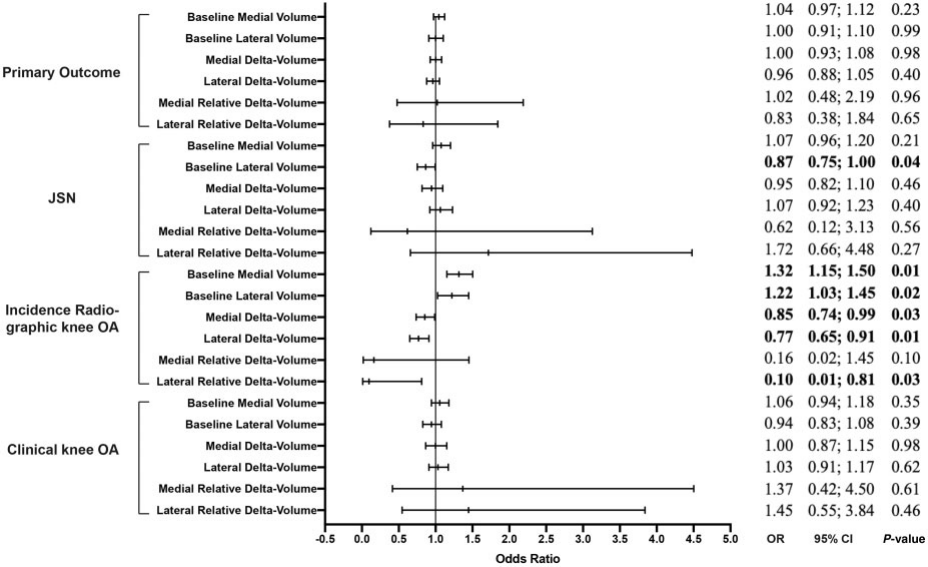
Association between baseline and delta meniscal volume and primary and secondary outcomes (baseline and 30 months) All odds ratios are adjusted for meniscal volume, BMI, age, knee injury and knee alignment, postmenopausal status, Heberden’s nodes, meniscal pathologies, extrusion, osteophytes and cartilage defects at baseline. OR: odds ratio; JSN medial (lateral): medial (lateral) joint space narrowing. OR>1 signify larger volume at baseline or growth of volume during follow-up. OR<1 signify lower volume at baseline or shrinkage of volume during follow-up.

Baseline medial and lateral volume were not significantly associated to the primary outcome [odds ratio (OR)=1.04; 95% CI: 0.97, 1.12 and OR=1.00; 95% CI: 0.91, 1.10]. Lateral meniscal volume (not medial) was significantly associated to lateral JSN (OR =0.87; 95% CI: 0.75, 1.00). Baseline medial and lateral volume were both significantly associated with incident radiographic knee OA (OR=1.32; 95% CI: 1.15, 1.50 and OR=1.22; 95% CI: 1.03, 1.45). Additional adjustments for intervention groups did not result in significant changes of the results (data not shown). The associations between all baseline meniscal volumes and incident clinical knee OA were not statistically significant (see Fig. 2).

### Longitudinal meniscal volume changes and knee OA development

All associations between meniscal delta-volume, relative delta-volume and the primary and secondary outcome measures are presented in Fig. 2. Neither medial nor lateral delta-volume were significantly associated with the primary outcome or medial/lateral JSN. Both medial and lateral delta-volume showed significant associations with incident radiographic knee OA (OR=0.85; 95% CI: 0.74, 0.99 and OR=0.77; 95% CI: 0.65, 0.91). Lateral relative delta-volume was significantly associated to incident radiographic knee OA (OR=0.10; 95% CI: 0.01, 0.81). The associations between all meniscal changes and incident clinical knee OA were not significant. Additional adjustments for intervention groups did not result in significant changes of the results (data not shown).

### Meniscal extrusion

**Fig. 3:**
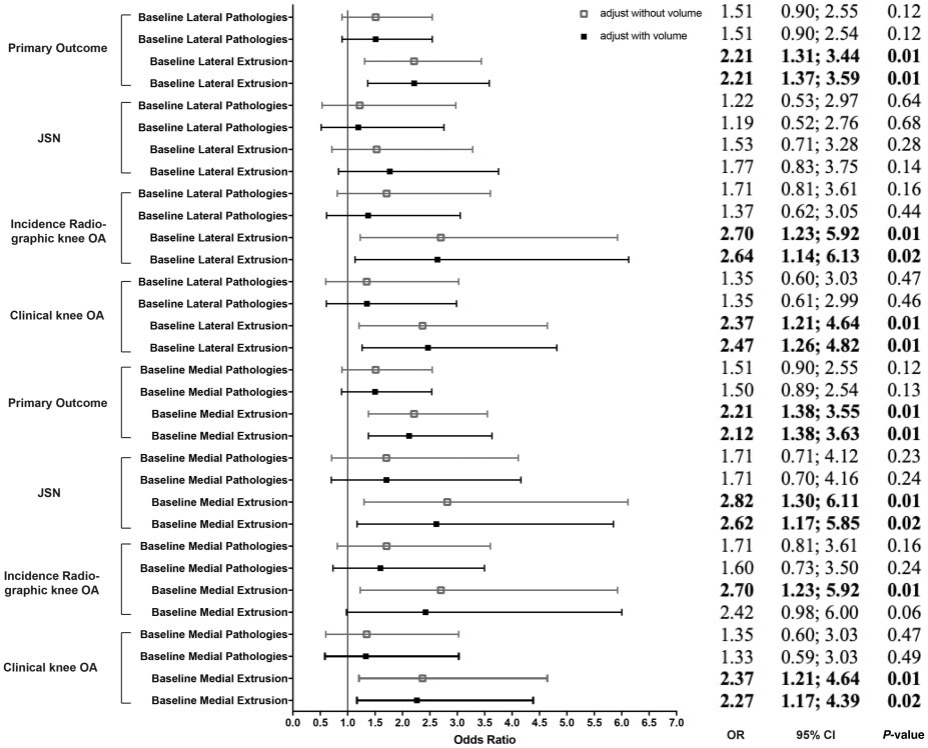
Association between baseline meniscal extrusion and primary and secondary outcomes, with and without adjustment for meniscal volume (baseline and 30 months) All odds ratios are adjusted for meniscal volume, BMI, age, knee injury and knee alignment, postmenopausal status, Heberden’s nodes, meniscal pathologies, extrusion, osteophytes and cartilage defects at baseline. OR: odds ratio; JSN medial (lateral): medial (lateral) joint space narrowing. Hollow square: adjusted without meniscal volume; solid square: adjusted with meniscal volume.

By comparing the association between meniscal extrusion and all outcomes with and without adjusting for baseline meniscal volume, we found the odds for OA development in knees with meniscal extrusion only changed marginally after additional adjustment for baseline meniscal volume (see Fig. 3).

## Discussion

In the present study we evaluated the association between the volume of the meniscus and its change over time and the development of knee OA in a high-risk group of overweight and obese women. We found that subjects with larger baseline volume (potentially suggestive for meniscus swelling) and a decrease of meniscal volume over time had a higher risk for incident radiographic knee OA. Only baseline lateral meniscal volume was associated with lateral JSN, while neither medial nor lateral meniscal volume were significantly related to incident clinical knee OA.

The meniscus is considered to be a protective structure by providing biomechanical support in a healthy knee joint. However, as our results indicate, both larger meniscal volume at baseline and the decrease of volume during FU may act as risk factors for the development of knee OA in overweight and obese women. Previously, Andrea *et al.* reported larger meniscal volume in the lateral meniscus body in knee OA subjects [13] and Wolfgang *et al.* found that menisci were thicker in OA knees and had a larger meniscal volume when compared with non-OA knees [8]. As individuals in the current study were free of clinical knee OA at baseline, the results suggest that swelling of the menisci may take place prior to the shrinkage of the menisci, along with the development of structural knee OA; similar to cartilage swelling that is reported to occur prior to cartilage degeneration [31, 32].

We found that meniscal volume was not significantly related to the incidence of clinical knee OA. This may be because the FU period was only 30 months, when clinical complaints like pain may not be observed yet in people free of symptoms and disease at baseline [17]. Other studies also concluded that structural features of OA (e.g. osteophytes) were more reliable than clinical symptoms as an early indication of knee OA, as pain is more commonly seen in higher grades of OA [33, 34]. As individuals with more severe radiographic OA features show an increased risk for the presence of knee pain [35], it is important to identify individuals at increased risk for radiographic knee OA, for example using meniscal volume as a predictive biomarker.

As greater baseline meniscal volume and decrease of volume during FU were associated to the incidence of K&L  ≥  2, which is defined by the combination of definite osteophytes and possible JSN, but not to JSN alone, we could further hypothesize that meniscal volume is related to osteophyte formation. As a consequence of meniscal volume change, mechanical stresses or soluble growth factors like insulin-like growth factor-1, fibroblast growth factor and bone morphogenetic protein or transforming growth factor-β may activate compensatory cartilage repair, which then induces the osteophyte formation [36–38].

According to previous studies and our current results, meniscal volume and meniscal extrusion are both independently associated to incidence of radiographic knee OA [11, 39]. There are several theories suggesting that meniscal volume and extrusion are interrelated. Wenger *et al.* suggested that meniscal extrusion could coexist with change in meniscal volume, possibly because the extruded part of the meniscus potentially swells as it becomes unloaded outside the joint margin [13]. Another hypothesis is that a swollen meniscus at baseline may be more vulnerable to becoming extruded, owing to its larger size. The displacement of the meniscus caused by both meniscal extrusion and swelling may alter the knee load distribution capacities, which could lead to osteophyte formation and cartilage loss. However, further research is needed to test these hypotheses.

There are some strengths and limitations to our study. By using MRI, we confirmed a quantitative biomarker of meniscal volume to be associated with the incidence of radiographic knee OA. This measurement potentially provides a tool to detect knee OA in overweight women, especially in the early phase of the disease. Early detection may help intervention since pre-OA is suggested to be a modifiable disease process [40]. Also, the change in meniscal volume during FU has the potential to become a surrogate end point. Moreover, our analyses make use of automatic segmentations of the meniscus, instead of manual segmentations, as it means the segmentations are objective and repeatable, which would make it more suitable for future clinical use. One limitation is that three different scanners were used throughout the cohort. However, the scanner type was only associated to meniscal volume, which was the exposure in the GEE models. The adjustment for scanner type should therefore be unnecessary [41]. Although there were different treatment groups in this cohort, additional adjustment for the treatment groups did not significantly affect the results (data not shown). Another limitation was the FU time of only 30 months, which might be relatively short for evaluating a degenerative disease, especially in subjects without symptoms at baseline. In this study, we did not indicate a cut-off value for meniscal volume in subjects with high risk of knee OA. Once meniscal volume is indisputably proven as a biomarker for knee OA development, new initiatives on valuable cut-off scores should be undertaken.

## Conclusion

As is known for cartilage volume, knees with higher baseline meniscal volume and a stronger decrease in meniscal volume over time are at increased risk for developing radiographic knee OA. Given the lack of a (reversed) association between meniscal measures and medial/lateral JSN, this suggests a relation with osteophyte growth, but this relation needs to be confirmed in future studies. Meniscal volume may function as a prognostic biomarker for future structural knee OA in overweight and obese women.
